# Linear array measurements of enhanced dynamic wedge and treatment planning system (TPS) calculation for 15 MV photon beam and comparison with electronic portal imaging device (EPID) measurements

**DOI:** 10.2478/v10019-010-0037-5

**Published:** 2010-09-09

**Authors:** Borislava Petrovic, Aleksandra Grzadziel, Laza Rutonjski, Krzysztof Slosarek

**Affiliations:** 1 Department of Radiotherapy, Institute of Oncology Vojvodina, Sremska Kamenica, Serbia; 2 Radiotherapy and Brachytherapy Planning Department, Comprehensive Cancer Centre, Maria Sklodowska Curie Memorial Institute, Gliwice, Poland

**Keywords:** enhanced dynamic wedge, linear array, EPID

## Abstract

**Introduction.:**

Enhanced dynamic wedges (EDW) are known to increase drastically the radiation therapy treatment efficiency. This paper has the aim to compare linear array measurements of EDW with the calculations of treatment planning system (TPS) and the electronic portal imaging device (EPID) for 15 MV photon energy.

**Materials and methods.:**

The range of different field sizes and wedge angles (for 15 MV photon beam) were measured by the linear chamber array CA 24 in Blue water phantom. The measurement conditions were applied to the calculations of the commercial treatment planning system XIO CMS v.4.2.0 using convolution algorithm. EPID measurements were done on EPID-focus distance of 100 cm, and beam parameters being the same as for CA24 measurements.

**Results:**

Both depth doses and profiles were measured. EDW linear array measurements of profiles to XIO CMS TPS calculation differ around 0.5%. Profiles in non-wedged direction and open field profiles practically do not differ. Percentage depth doses (PDDs) for all EDW measurements show the difference of not more than 0.2%, while the open field PDD is almost the same as EDW PDD. Wedge factors for 60 deg wedge angle were also examined, and the difference is up to 4%. EPID to linear array differs up to 5%.

**Conclusions:**

The implementation of EDW in radiation therapy treatments provides clinicians with an effective tool for the conformal radiotherapy treatment planning. If modelling of EDW beam in TPS is done correctly, a very good agreement between measurements and calculation is obtained, but EPID cannot be used for reference measurements.

## Introduction

Mechanical wedge filters (hard wedges) are often used in the treatment planning as compensators of dose inhomogeneities in the photon therapy. Nowadays, they are often replaced by Enhanced Dynamic Wedge (EDW). EDW is a technical solution of Varian Medical Systems, but also other manufactureres have solutions which achieve the same result (Elekta- omni wedge, Siemens- virtual wedge). The EDW technique achieves wedge-shaped dose distributions by the computer-controlled movement of one of the collimator jaws under the simultaneous adjustment of dose rate and speed of the moving jaw. The relationship between the number of delivered monitor units and the position of the moving jaw is governed by lookup tables referred to as ”Segmented Treatment Tables” (STT). The EDW provides seven wedge angles (10°, 15°, 20°, 25°, 30°, 45°, and 60°) for both symmetric and asymmetric field sizes. The upper independent jaws, assigned to as Y1 and Y2, can travel from a full open position to 10 cm across the central axis, thus allowing field sizes up to 30 cm along the wedged direction. Two wedge orientations are available: Y1-IN and Y2-OUT, indicating the moving jaw. The EDW needs only one reference STT for each photon energy. This so called ”Golden” STT represents the full field width of 30 cm and a wedge angle of 60°. Intermediate wedge angles can be derived by means of weighted averaging of an ”open field STT” and the Golden STT (ratio of tangens method). The individualized treatment STT is then obtained by the truncation to the desired field size and normalization so that the final number of monitor units is the total number of monitor units needed to deliver a certain dose to the reference point. These individualized STTs are created automatically by the linac computer, as the operator types in the energy, wedge angle, monitor units, etc. In order to deliver a dynamically wedged field, the length of the treatment field is divided into 20 segments, and the speed of the moving jaw and the dose rate within each segment are controlled based on a calculated segmented treatment table (STT) generated by the linear accelerator computer.

The implementation of dynamic wedges in the various radiation therapy planning (RTP) systems has already been described.^1^,^2^ As with any other commissioning activity, great care must be taken to ensure that enhanced dynamic wedges are correctly modelled in the treatment planning system. To directly verify the computational accuracy of a treatment planning system, measurements need to be made with the accelerator setup to the same identical specifications as already planned.^3^

This work was aimed to verify EDW (described in details in literature)^4^ in the treatment planning system (TPS) and use patient set up equipment to compare dosimetrical and calculation results with electronic portal imaging device (EPID) measurements. In addition, comparison with hard wedges was also presented.

The electronic portal imaging device is very sophisticated gadget, accessory at the stand of the accelerator, which has an amorphous silicon detector remaining resistant to irradiation after the application of very high doses, and has certain dosimetrical characteristics which were also investigated here but also well described in literature.^5^–^10^

## Materials and methods

### Linear array CA24 measurements

The measurement of enhanced dynamic wedge profiles using a linear chamber array requires the integration of the dose during the entire exposure at each point of measurement. It was done by the CA 24 Scanditronix Welhofer, and two electrometers, MD 240 and CU 500E, connected to the PC and OmniPro 6.2A software. The linear array CA 24 consists of 23 ionization chambers, the volume of each is 0.147 cm^3^, diameter 0.6 cm and active length 0.33 cm. The each two neighbouring chambers are placed on 2 cm distance, and their long axes are parallel to the central axis of the beam. They are mounted to the holder of the Blue water phantom. The main feature of this linear array is that the profiles are measured directly in the water, under the same conditions as measurements of the open field profiles or mechanical wedged field profiles.

The beam data was collected according to the guidelines provided by Varian^5^,^6^. This consists of measurements of cross profiles and depth dose curves for the maximum (60°) and at least one intermediate wedge angle, in addition to measurements of the output factors.

The calculated percentage depth dose curves (PDDs) and profiles were compared with measured data for 15 MV photons at a Varian Clinac 2100C. Square field sizes ranging from 4*x*4 cm^2^ to 20*x*20 cm^2^ were evaluated with measurements of PDDs and profile curves on few depths (build up, 5 cm, 10 cm, and 20 cm).

### EPID measurements

The features of EPID are described well in the literature.^7^–^13^ Portal imager aS1000 was positioned on a source to skin distance (source-EPID surface distance- SSD) 100 cm (not on standard 140 cm). The standard calibration procedure was then applied under this condition.

The EDW fields of 4 cm × 4 cm, 10 cm ×10 cm, 15 cm × 15 cm, and 20 cm × 20 cm were imaged (with the usage of EPID portal dosimetry mode) for the wedge angles of 15 deg, 30 deg, 45 deg and 60 deg, with the collimator orientation and movement as for CA 24 measurements. The collimator orientation for all measurements was 90 degrees and Y1-IN wedge orientation (*Y1* being the dynamic jaw).

Linearity of the pixel response with dose was checked, followed by field measurements.

The image acquired by EPID, which results from each EDW field irradiation, is 2D image, with the different pixel values and is closely related to the intensity map of the EDW field. The pixel values carry information about the intensity of the signal within the pixel area. Pixels lying on lines crossing the central axis pixel are creating in plane and cross plane profiles. One profile is in the direction of the moving jaw, creating the wedged distribution, and another one is the perpendicular to the direction of the moving jaw. Other pixels are lying off axis, and can be used to create 3D image of a wedged field.

In order to extract useful information about the profiles, the central axis pixel value is assigned value 100. All other pixels got then a relative value, depending on the ratio of the original pixel value on central axis, and elsewhere in plane and cross plane profiles. The series of relative pixel values on both profiles creates profiles comparable to other methods of measurements.

### External beam treatment planning calculations

The treatment planning system used for this purpose was XIO CMS v. 4.2.0, convolution algorithm. Virtual phantom of the size of the big Blue phantom (used for measurements in water), was defined in the TPS, and the electron density of water assigned to the inner space of the phantom. The EDW beam was created with the collimator and gantry orientation as in water and EPID measurements, and appropriate field size, wedge angle, weight point definition, normalization, etc, imitating the measurements under real conditions in water. The resulting calculated plan was analyzed taking into consideration the depth dose curve and profiles on determined depths (build up, 5 cm, 10 cm and 20 cm). Dose values were read from the Dose Profile in the menu of the treatment planning space of XIO, on 5 mm distance along the profile of the field.

These calculated profiles, as well as the profiles obtained by CA 24, and EPID, were compared to the profiles of hard wedges obtained using Blue phantom and CC13 ionization chambers, collected upon commissioning and acceptance tests of this linear accelerator.

### Hard wedges measurements and open field measurements

The measured data of open fields and for hard wedges, collected during commissioning and acceptance tests of the Varian 2100C linac were used for this study. Only additional measurements for the field 4×4 cm^2^ were collected during this survey for all wedge angles and depths, since Varian recommendations for commissioning do not include this field size as mandatory.

## Results

### Percentage depth doses

The percentage depth dose curves of the open fields (measured by CC13 chambers), hard wedged fields (measured also by CC13 chambers), EDW fields (measured by linear array CA 24- PDD values extracted from profiles) and calculated by XIO, were compared.

Generally speaking, the PDDs of open fields and EDW fields do not differ more than 0.5%.

**FIGURE 1:**
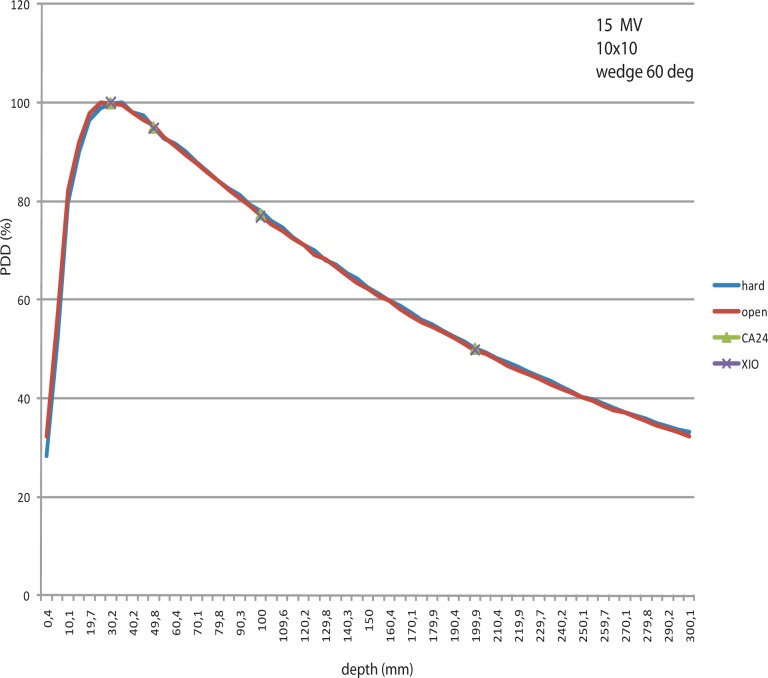
PDD of 10 cm × 10 cm field, 15 MV, wedge 60 deg.

PDDs of open fields have a higher surface dose than the PDDs of hard wedged fields (dose extrapolated to the surface of water- 0 cm depth) (Figure 1). This comes from the beam hardening under the mechanical wedge. The beam hardening effect is also clearly visible on the tail of the PDD curve of the mechanical wedge and gives the difference of around 2%.

PDDs generated from profiles measured by CA 24 and calculated by XIO are practically identical (result of modelling the EDW in TPS).

The PDDs with EPID could not be obtained at this stage, since only measurements in build up were possible.

### Profile measurements

#### EPID profiles in build up compared to linear array measurements in build up

Profiles were obtained in direction of the moving jaw, showing the wedged shaped distribution. EDW profiles obtained by EPID in comparison with the same measured by the linear array differ around 1%, max up to 2%, within the field (Table 1). At the edges of the fields, the EPID profiles were having a larger gradient (dose fall down) than the profiles obtained by other methods. This applies to all wedge angles.

**FIGURE 2:**
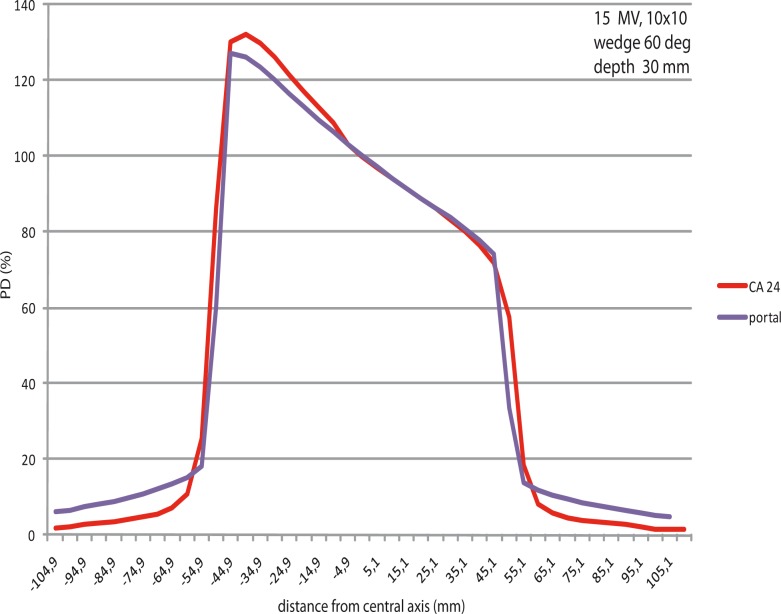
EPID profile *vs* CA24 profile, 10 cm x10 cm field, wedge 60 deg.

A dose measured by EPID outside the field (peripheral dose) was much larger than the one measured by CA 24 linear array. This is characteristic for all angles and for all field sizes. (Figure 2)

### Profiles measured by EPID in comparison to open beam profiles measured by ionization chamber

**FIGURE 3:**
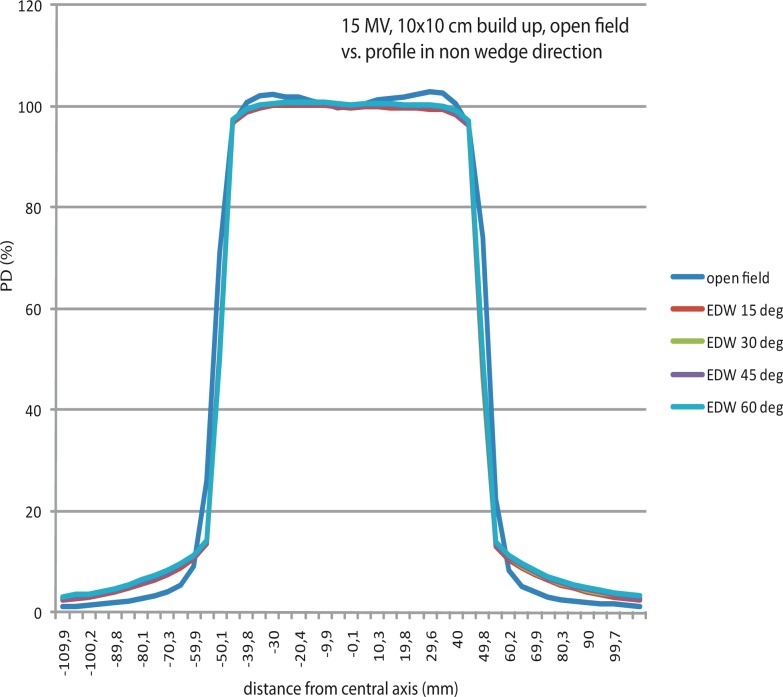
Open field profiles overlap with the EDW profiles in non wedged direction (example is 10 cm × 10 cm field).

EDW profiles imaged by EPID in the perpendicular direction to the movement of the jaw, were also examined, and compared to the open field profiles, which were measured during commissioning of the machine, by CC13 ionization chambers. A very good agreement was found (Table 2, Figure 3). This is not the case with the profiles of hard wedged fields, measured also in the non-wedged direction, where the interaction of the beam with the material of the hard wedge (beam hardening effect), influences the shape of the profile (a hard wedged profile demonstrates a decrease in dose at the field edges in comparison with the EDW and open field profile in non wedged direction).

### Profiles of EDW field measured by linear array in comparison of hard wedges profiles measured by ionization chambers

**FIGURE 4:**
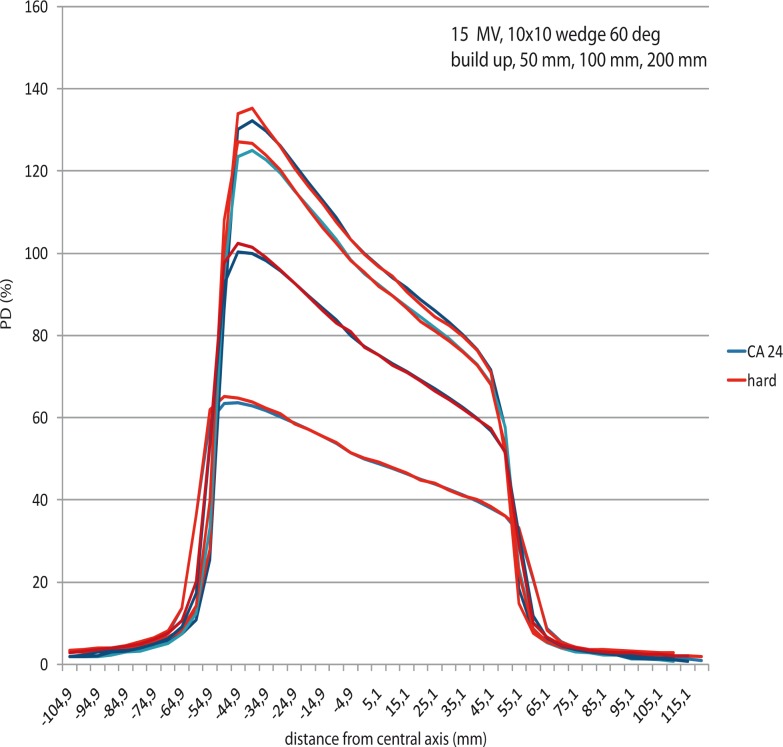
CA 24 profiles in comparison with hard wedge profiles, 10 cm × 10 cm field, 60 deg wedge.

EDW linear array profiles to hard wedges do differ more in all cases, but that was expected due to the physical differences of two techniques (Figure 4)

### Profiles of EDW field measured by linear array in comparison to the calculation of XIO CMS TPS

In most cases, the dose values on profiles differ around 0.5%, within the field, while outside the field it seems that XIO underestimates the peripheral doses by factor of 2.

### EDW wedge factors

EDW wedge factors are the strong functions of the field size. This is proved by the measurements of wedge factors of EDW fields, and by the calculation of WF in the treatment planning system. This, of course, does not apply to the hard wedge whose dependence of the field size is almost negligible. This is due to the fact that mechanical wedges are always placed in the same position on the tray of the accelerator, and because the central beam always passes through the same thickness of the wedge, it does not matter what the field size is actually set (Table 3).

## Discussion

For the quality assurance (QA) in radiotherapy we can use in *vivo* or *in vitro* methods with phantoms.^14^ The second one can be used for for routine QA or for reference measurements. The basic conclusion of our study would be that EPID aS1000 can be used for the routine QA and for EDW verification, but not for commissioning, only for regular QA checks. The conclusion would also be that the implemented dose calculation algorithm well describes the EDW treatment.

The peripheral dose of EDW field is half the dose of the hard wedged field. The reason for that lies in scatter outside the hard wedged field, due to the interaction of the beam with the material of the mechanical wedge. Clinically, this is an advantage of EDW wedged field. The wedge angle is better preserved for EDW than for hard wedges at all depths.

The profile dose measured by EPID outside the field (peripheral dose) was much larger than the one measured by the CA 24 linear array. This is characteristic of all angles and for all field sizes. The reason for that as explained in the literature, might be due to the difference in absorption of low energy photons which appears in the material of the high Z. Spectrum of the photons is changed with the distance from the central axis, and region outside the field has only a scatter radiation. That is why the difference in profiles outside the field can be assumed to come from the difference of low energy photons of other dosimetrical methods and sensitive material of EPID detectors.

**FIGURE 5:**
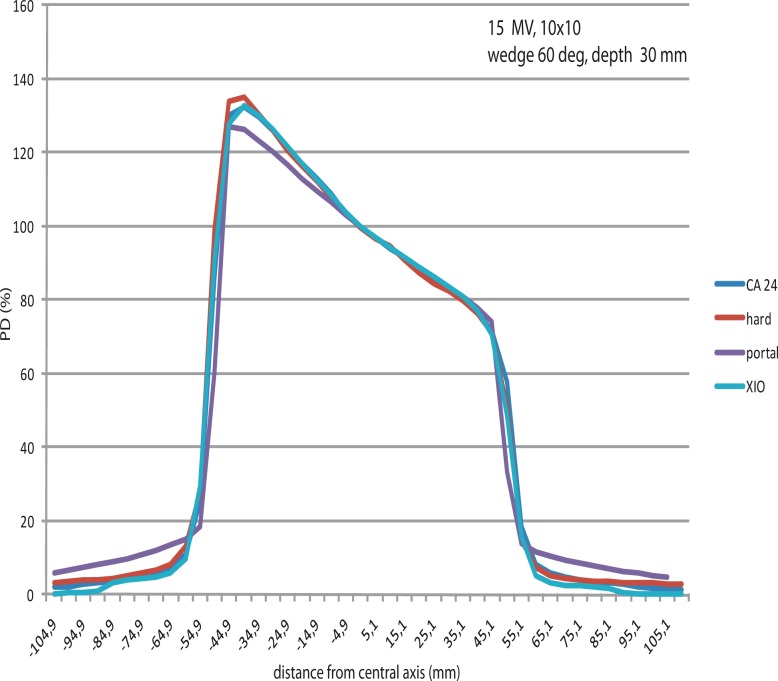
15 MV profiles for a 10 cm ×10 cm field, 60 deg wedge, build up (four methods of measurements and calculations).

Practically, all measurement techniques of EDW give very satisfactory results in terms of the agreement within PDDs and profiles (Figure 5). Still, standard dosimetric measurements cannot be underestimated, and EPID implemented as verification tool in terms of implementation of a new technique in the department.

## Figures and Tables

**TABLE 1:** EDW profile measurements CA 24 in build up versus EPID

**4×4 cm^2^, 15deg (field edge 2 cm)**			**10×10 cm^2^, 30deg (field edge 5 cm)**			**15×15 cm^2^, 45deg (field edge 7.5 cm)**			**20×20 cm^2^, 60deg, (field edge 10 cm)**		

**Position of detector in relation to Central axis (mm)**	**CA 24**	**EPID**	**Position of detector in relation to Central axis (mm)**	**CA 24**	**EPID**	**Position of detector in relation to Central axis (mm)**	**CA 24**	**EPID**	**Position of detector in relation to Central axis (mm)**	**CA 24**	**EPID**
−50	1.03	3.01	−100	1.83	6.17	−120	3.86	10.00	−140	6.98	16.08
−45	1.87	3.53	−95	2.55	6.78	−115	4.33	10.87	−135	7.76	17.06
−40	1.9	3.97	−90	2.82	7.47	−110	5.13	11.87	−130	8.67	18.67
−35	2.84	4.49	−85	3.37	8.27	−105	5.66	13.18	−125	9.87	20.40
−30	4.78	5.32	−80	3.71	9.20	−100	6.31	14.49	−120	11.56	22.07
−25	12.02	7.37	−75	4.36	10.13	−95	7.37	15.71	−115	14.08	24.09
−20	50.2	47.84	−70	4.85	11.14	−90	9.49	17.28	−110	21.05	25.99
−15	93	99.39	−65	6.61	12.39	−85	14.53	19.16	−105	65.42	30.55
−10	100.19	100.74	−60	10.25	13.92	−80	39	22.52	−100	160.61	92.80
−5	100.12	100.44	−55	25.45	16.75	−75	107.34	59.80	−95	185.95	166.63
0	100	100.00	−50	79.05	52.91	−70	130.84	124.18	−90	186.12	166.80
5	99.59	99.65	−45	108.59	107.10	−65	133.61	125.23	−85	181.62	164.32
10	98.68	99.00	−40	111.69	108.19	−60	132.46	124.01	−80	176.63	160.58
15	94.37	96.90	−35	111.59	107.67	−55	130.4	122.30	−75	171.42	156.95
20	67.12	44.70	−30	110.35	106.74	−50	127.98	120.69	−70	166.64	152.97
25	19.13	7.28	−25	108.99	105.73	−45	125.24	118.90	−65	161.35	149.40
30	6.51	5.32	−20	107.33	104.52	−40	122.26	116.85	−60	156.44	145.25
35	2.93	4.58	−15	105.6	103.27	−35	119.87	114.80	−55	151.61	141.50
40	2.38	4.06	−10	103.94	102.38	−30	117.16	112.61	−50	146.82	137.41
45	1.87	3.58	−5	101.19	101.13	−25	113.98	110.34	−45	141.25	133.43
50	1.85	3.10	0	99.98	100.00	−20	111.23	108.03	−40	136.55	129.28
			5	99.28	99.19	−15	108.43	106.11	−35	131.67	125.48
			10	98.76	98.43	−10	105.69	104.02	−30	127.09	121.50
			15	98.32	97.38	−5	102.07	101.92	−25	122.26	117.46
			20	97.72	96.61	0	99.91	100.00	−20	117.6	113.55
			25	96.96	95.88	5	98.5	98.43	−15	112.98	110.32
			30	96.11	95.04	10	97.11	97.03	−10	108.93	106.80
			35	94.97	93.95	15	95.96	95.37	−5	103.32	103.23
			40	93.17	92.74	20	94.61	93.98	0	99.93	100.00
			45	88.98	90.27	25	93.34	92.84	5	96.96	97.06
			50	70.98	38.82	30	91.98	91.58	10	94.28	94.41
			55	22.68	14.97	35	90.57	90.22	15	91.59	91.76
			60	9.09	12.63	40	89.04	88.83	20	89.04	89.16
			65	5.97	11.22	45	87.5	87.47	25	86.52	86.63
			70	5	10.21	50	85.97	85.68	30	83.75	84.44
			75	4.13	9.20	55	84.05	84.20	35	81.32	82.19
			80	3.34	8.31	60	82.11	82.15	40	78.55	79.71
			85	3.29	7.59	65	79.78	80.18	45	76.2	77.46
			90	2.65	6.86	70	76.51	76.87	50	73.56	74.99
			95	2.35	6.13	75	63.15	34.00	55	70.96	72.62
			100	1.63	5.65	80	26.21	16.24	60	68.4	70.32
						85	10.32	14.27	65	65.96	67.95
						90	7.25	12.92	70	63.53	65.59
						95	4.92	11.79	75	61.05	63.17
						100	4.19	10.74	80	58.53	60.81
						105	4.04	9.86	85	56.34	58.67
						110	3.48	9.04	90	54.11	56.08
						115	3.01	8.25	95	49.82	52.97
						120	2.74	7.64	100	43.59	24.67
									105	21.59	14.52
									110	8.34	13.08
									115	5.9	11.99
									120	4.91	11.24
									125	4.5	10.43
									130	3.84	9.63
									135	3.38	9.11
									140	3.14	8.70

**TABLE 2:** Open field profiles in 3 cm build up vs EPID profiles in direction perpendicular to the movement of Y jaw, 10×10 cm2 field

**Crossline (mm)**	**Open**	**15deg EDW**	**30 deg EDW**	**45deg EDW**	**60deg EDW**
−110	1.3	2.8	2.8	2.9	2.9
−105	1.3	3.1	3.1	3.2	3.2
−100	1.5	3.2	3.2	3.2	3.3
−95	1.7	3.8	3.8	3.8	3.9
−90	2.0	4.3	4.3	4.3	4.4
−85	2.2	5.1	5.1	5.1	5.2
−80	2.7	6.0	6.0	6.0	6.1
−75	3.3	6.7	6.8	6.8	6.9
−70	4.0	7.8	7.9	7.9	8.0
−65	5.4	9.3	9.3	9.3	9.4
−60	9.2	11.0	11.0	11.0	11.1
−55	26.1	13.9	14.0	14.0	14.0
−50	71.2	51.0	51.5	51.6	49.7
−45	96.5	97.1	97.3	97.1	97.0
−40	100.9	99.2	99.3	99.2	99.1
−35	102.2	100.1	100.2	100.1	100.0
−30	102.3	100.5	100.6	100.5	100.4
−25	101.9	100.6	100.6	100.6	100.5
−20	101.8	100.5	100.6	100.6	100.5
−15	101.1	100.7	100.7	100.6	100.6
−10	100.5	100.5	100.6	100.6	100.5
−5	99.7	100.4	100.4	100.4	100.3
0	100.0	100.0	100.0	100.0	100.0
5	100.6	100.2	100.4	100.3	100.3
10	101.2	100.2	100.2	100.2	100.2
15	101.5	100.1	100.2	100.2	100.2
20	101.9	100.0	100.1	100.0	100.0
25	102.5	100.0	100.1	100.1	100.0
30	102.9	99.8	100.0	99.9	100.0
35	102.6	99.6	99.7	99.7	99.7
40	100.5	98.8	98.8	98.8	98.9
45	96.2	96.6	96.7	96.6	96.8
50	74.1	48.4	46.2	48.3	50.1
55	22.5	13.4	13.4	13.5	13.6
60	8.3	10.8	10.9	11.0	11.0
65	5.1	9.2	9.2	9.3	9.4
70	4.1	7.8	7.9	8.0	8.0
75	3.1	6.7	6.8	6.8	6.9
80	2.6	5.8	5.8	5.8	6.0
85	2.3	5.1	5.1	5.2	5.3
90	1.9	4.4	4.5	4.5	4.6
95	1.6	3.9	3.9	4.0	4.1
100	1.6	3.4	3.5	3.5	3.5
105	1.4	3.1	3.1	3.2	3.2
110	1.2	2.9	2.9	2.9	3.0

**TABLE 3:** WF measured for the angle of 60°, and field sizes 4×4 cm2, 10×10 cm2, 15×15 cm2, 20×20 cm2, 30×30 cm2 using the energy of 15M

**X(cm)**	**Y1 (cm)**	**Y2(cm)**	**Measured WF**	**TPS WF**	**Hard wedge WF**
4	2	2	0.892	0.882	0.431
10	5	5	0.713	0.689	0.437
15	7.5	7.5	0.596	0.575	0.444
20	10	10	0.499	0.483	n/a
30	20	10	0.343	0.345	n/a
